# Development and validation of the manual disease activity score (MDAS) for rheumatoid arthritis

**DOI:** 10.1515/rir-2025-0031

**Published:** 2025-12-27

**Authors:** Babur Salim, Amjad Nasim, Saira Jahan, Shahida Perveen, Saba Samreen, Haris Gul, Ali Afzal, Noor ul Ain Rizwan, Maryam Ahmed, Saira Yasmin

**Affiliations:** Rheumatology department, Fauji Foundation Hospital, Rawalpindi, Pakistan; Foundation University, Islamabad, Pakistan; Fauji Foundation Hospital, Pain Clinic, Rawalpindi, Pakistan; Armed Forces Institute of Rehabilitation Medicine, Rawalpindi, Pakistan

Dear Editor,

Rheumatoid arthritis (RA) is a chronic, autoimmune disease that primarily effects small synovial joints ^[1]^ with potential systemic involvement.^[2]^ Approximately 0.5–1% of individual worldwide is affected, with a higher prevalence in females and in older adults.^[3]^

Regular and accurate assessment of disease activity is crucial in managing RA, as it assists in guiding the treatment choices and aims to attain remission.^[4]^

To facilitate this approach, many composite disease activity scores have been developed, validated and widely accepted in clinical settings. These are the Disease Activity Score 28 (DAS28), Routine Assessment of Patient Index Data 3 (RAPID3), Clinical Disease Activity Index (CDAI), and Simplified Disease Activity Index (SDAI).^[5,6]^ These tools integrate clinical joint assessments, patient-reported outcomes, and blood markers such as erythrocyte sedimentation rate (ESR) or high sensitivity C-reactive protein (hs-CRP).^[7]^ Although they are highly informative and have been validated in multiple cohorts, their regular use can sometimes be challenging in clinical settings. The need for laboratory testings causes delays in clinical decision-making, while complex formulas may impede their implementation in outpatient settings. Additionally, DAS28 includes 28 joints, excluding those in the feet and ankles—particularly the metatarsophalangeal (MTP) joints—which are often affected in early and active RA.

This exclusion might result to underestimation of disease activity, specifically in patients with significant foot involvement.

Numerous tools comprise shoulder joints,^[8,9]^ which can be sometimes be difficult to assess particularly for swelling because of the adjacent muscles and the variability in patient’ anatomy. At times, patients may feel uneasy or culturally sensitive to expose their shoulders, which can pose a challenge during clinical assessment. Improper evaluation of shoulder joints decreases inter-rater reliability and introduces subjectivity into clinical assessment. There is thus a clear need for a quicker, simpler, practical, accessible, and objective tool that can provide reliable assessments of disease activity in RA without relying on laboratory investigations.

In order to address these challenges, we propose the development of a Manual Disease Activity Score (MDAS) annexure A (Supplementary Material)—a simplified, clinician-administered tool that relies exclusively on joint examination, physician global assessment (PhGA) and patient-reported outcomes (PtGA), without the need for laboratory investigations. MDAS aims to preserve the clinical accuracy while improving practicality, enhancing feasibility and accessibility in various healthcare settings. It introduces the MTP joints to improve sensitivity and disease detection while excluding the shoulder joints to prevent variability in assessment. Our study goal is to offer a practical and reliable alternative to existing tools that can be widely used in outpatient clinics particularly in low-resource environments.

Additionally, MDAS introduces metacarpophalangeal joint (MCP) & MTP squeeze test instead of individually assessing each joint. This is based on the rationale that the clinical decision—such as whether to escalate treatment—remains the same regardless of whether one or multiple joints are inflamed. Incorporating the squeeze tests streamlines the assessment process, making it quicker and more practical in routine clinical settings. The objective was to develop and validate the MDAS in RA by measuring its reliability, validity, correlation and cohen’s *kappa* values with existing standard indices of RA disease activity. By offering a more inclusive and easily applicable tool, MDAS has the potential to enhance clinical decision-making and improve accessibility in RA care, determine the severity of disease and promote equitable RA care delivery regardless of setting or resource availability.

A cross-sectional validation study was executed to develop and validate the *Manual Disease Activity Score (MDAS)* for assessing the disease activity in RA patients. The study was carried out from July 2024 to June 2025 at rheumatology outpatient clinics Fauji Foundation Hospital, Rawalpindi, Pakistan, following ethical approval from the Institutional Review Board of Fauji Foundation Hospital, Rawalpindi, Pakistan. Patients with rheumatoid arthritis aged 18 years and above who fulfilled the 2010 American College of Rheumatology/European League Against Rheumatism (ACR/EULAR) classification criteria^[10]^ for rheumatoid arthritis were enlisted. Patients with systemic illness or other inflammatory arthritis were excluded to ensure the validity and reliability of the tool. The MDAS was proposed as a simple, clinician-administered, objective assessment tool to assess the disease activity based on manual palpation of following joints: MCP joints (MCP Squeeze test), MTP joints (MTP squeeze test), Knees, Wrists and Elbows. Each joint was palpated or squeezed by applying moderate pressure to detect pain, tenderness, or swelling. A binary scoring system was used: Tenderness with or without swelling: Score = 1, Absence of symptoms: Score= 0. The cumulative joint score was then combined with the PhGA in which Clinician-graded overall disease activity on a 0–10 numeric scale and Patient Global Assessment (PGA) in which Patient-graded perception of their disease activity on a 0–10 scale. The MDAS will include three parameters:(1) Joint synovitis score (0–10), (2) Physician Global Assessment (0–10), and (3) Patient Global Assessment (0–10). The final score will be calculated by summing the three components that reflects overall disease activity. To assess validity, MDAS scores were compared with two established disease activity indices *i.e*. RAPID3 (Routine Assessment of Patient Index Data) and CDAI (Clinical Disease Activity Index). These tools are frequently administered during the clinical evaluation. Descriptive statistics were applied to summarize demographic and clinical data. Pearson’s correlation coefficient was calculated to assess the relationship between MDAS and the comparator indices *i.e*. RAPID3 and CDAI. Cronbach’s alpha was used to evaluate the internal consistency and reliability of the MDAS tool. A *P*-value < 0.05 was considered statistically significant. The agreement between MDAS with CDAI and RAPID3 was measured by Cohen’s Kappa statistics. Data were analysed using SPSS version 23.0.

Out of the total 151 patients enrolled in the study, 84.1% were female and 15.9% were male with mean age 49.52 years with 28 (18.5%) were in remission, 41 (27.2%) had low disease activity, another 28 (18.5%) presented with moderate disease activity, while 54 (35.8%) exhibited high disease activity.

Convergent Validity and Internal Consistency of MDAS with CDAI and RAPID3 has been shown in Table 1. The agreement between the newly developed MDAS with established rheumatoid arthritis disease activity indices — CDAI and RAPID3 — was measured using Cohen’s Kappa statistics showing in Table 1. Based on agreement statistical analysis using cohen’s *kappa* with CDAI and RAPID3, the MDAS classifies disease activity in RA into four levels: remission, low disease activity, moderate disease activity, and high disease activity as shown in Table 2.

**Table 1:** Internal consistency, convergent validity, and agreement of MDAS with standard rheumatoid arthritis indices

Convergent Validity and Internal Consistency of MDAS with CDAI and RAPID3				
**Analysis Type**	**Statistic**	**CDAI**	**RAPID3**	**Remarks**
Convergent Validity	Pearson Correlation (*r*)	0.852	0.884	Strong positive correlation (*P* < 0.001)
	*P*-value	< 0.001	< 0.001	-
Internal Consistency	Cronbach’s Alpha	-	0.910	Excellent internal consistency
**Agreement Between MDAS and Standard RA Disease Activity Indices (Cohen’s Kappa Analysis)**				

**Comparison**	**Cohen’s Kappa (κ)**	**Strength of Agreement**		**Significance**
MDAS *vs*. RAPID3	0.607	Substantial		*P* < 0.001
MDAS *vs*. CDAI	0.550	Moderate		*P* < 0.001

**Table 2:** Rheumatoid arthritis disease activity categories based on MDAS interpretation

MDAS Score	Interpretation
0 – 4	Remission
5 – 9	Low Disease Activity
10 – 16	Moderate Disease Activity
> 16	High Disease Activity

Given its simplicity and time-efficiency, MDAS has strong potential for integration into routine rheumatology practice, complementing existing tools like CDAI and RAPID3.

Limitations of this study include the relatively small sample size, which may affect the generalizability of the findings. Additionally, the study population was predominantly female, which could introduce gender-related bias. Interphalangeal (IP) joints were excluded from the assessment; however, this did not significantly impact the reliability or validity of the tool, as substantial agreement was observed with established measures such as the CDAI and RAPID3. Future studies are recommended to include larger and more diverse populations, as well as to adopt a longitudinal approach to evaluate the tool’s sensitivity to changes in disease activity over time.

The Manual Disease Activity Score is a valid and reliable tool for assessing disease activity in rheumatoid arthritis. It shows strong agreement with established tools and establishes high internal consistency. Its simplicity and practicality make it suitable for routine clinical use, especially in settings where time or resources are limited.

## Supplementary Material

Supplementary Material Details
