# Supplementary material for: Development and validation of the manual disease activity score (MDAS) for rheumatoid arthritis
Source: Rheumatol Immunol Res. 2025 Dec 27;6(4):246–9. doi: 10.1515/rir-2025-0031 (PMC12743266; doi:10.1515/rir-2025-0031)
Supplement: Supplementary file 1 — Supplementary Material Details [file rir-2025-0031_sm.pdf]

Supplementary Material

Annexure-A

Manual Disease Activity Score (MDAS) –Tool for Rheumatoid Arthritis

A Tool for Assessing Rheumatoid Arthritis Disease Activity in Clinical Settings

---

Instructions for Use:

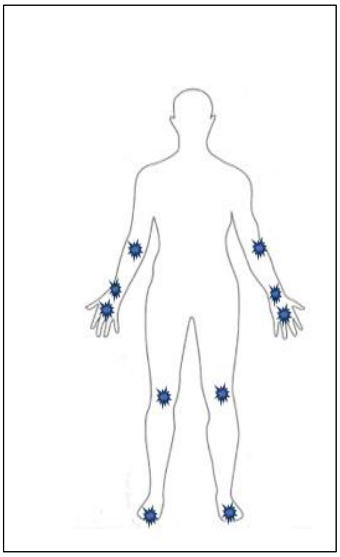

Physician should assess the following joints by palpation. Mark **1** if there is tenderness, swelling, or pain. Mark **0** if absent. Then sum the joint scores. Add Patient and Physician Global Assessments to calculate the total MDAS score.

**Joints**	**Tender? (With or without Swelling** (0 or 1)
Metacarpophalangeal-R	
Metacarpophalangeal-L	
Wrist-R	
Wrist-L	
Elbow-R	
Elbow-L	
Knee-R	
Knee-L	

Metatarsophalangeal-R	
Metatarsophalangeal-L	

Joint Score (sum, out of 10):

---

2. Patient Global Assessment (PGA)

Patient rates their overall disease activity on a scale from **0 (no disease activity)** to **10 (very severe disease activity)** :

PGA Score:

---

3. Physician Global Assessment (PhGA)

Physician rates overall disease activity on a scale from **0 (no disease activity)** to **10 (very severe disease activity)**:

PhGA Score:

Total MDAS Score

MDAS Total = Joint Score + PGA + PhGA

Total MDAS Score (0–30):

Disease Activity Categories

**MDAS Score**	**Interpretation**
0 – 4	Remission
5 – 9	Low Disease Activity
10 – 16	Moderate Disease Activity

> 16	High Disease Activity

This tool was developed and validated by Professor Dr. Babur Salim, Fauji Foundation Hospital, Rawalpindi, Foundation University, Islamabad.

Contact: babursalim@yahoo.com

Note: The MDAS is a rapid clinical tool to support real-time assessment of disease activity in RA. It does not require laboratory tests.
